# CDK12 antagonizes a viral suppressor of RNAi to modulate antiviral RNAi in *Drosophila*

**DOI:** 10.1128/mbio.02868-24

**Published:** 2024-11-27

**Authors:** Liqin Zhang, Yu Liang, Jiayu Qin, Chen Liu, Mengwei Shang, Xiaoming Sun

**Affiliations:** 1 Zhejiang Key Laboratory of Medical Epigenetics, Department of Immunology and Pathogen Biology, School of Basic Medical Sciences, Hangzhou Normal University, Hangzhou, China; 2 Department of Biochemistry and Molecular Biology, School of Basic Medical Sciences, Hangzhou Normal University, Hangzhou, China; 3 State Key Laboratory for Diagnosis and Treatment of Infectious Diseases, The First Affiliated Hospital, College of Medicine, Zhejiang University, Hangzhou, China; University of California at Riverside, Riverside, California, USA

**Keywords:** RNAi, viral suppressors of RNAi, B2, CDK12, counter-counter-defense

## Abstract

The primary antiviral immunity in insects is mediated by the RNA interference (RNAi) pathway. To counteract this antiviral RNAi response, viruses employ virulence factors known as viral suppressors of RNAi (VSR). The question of whether host factors can activate a counter-counter-defense mechanism to cope with VSR-mediated RNA silencing suppression remains unanswered. In this study, cyclin-dependent kinase 12 (CDK12) was identified to interact with B2, a VSR of Flock House virus (FHV), and the critical amino acids responsible for dsRNA binding and dimerization in B2 were essential for this interaction. Silencing of CDK12 facilitated FHV RNA accumulation only in the context of B2, not for FHVΔB2. Notably, CDK12 abrogated the RNAi suppression exerted by B2. Furthermore, the knockdown of CDK12 inhibited the production of vsiRNAs in FHV-infected *Drosophila* cells. This study revealed that CDK12 mediated a counter-counter-defense strategy against VSR, thereby enhancing antiviral RNAi immunity in *Drosophila*.

**IMPORTANCE:**

The arms race between virus and host immunity is never-ending. This study enhances our understanding of antiviral defenses in insects by uncovering a novel counter-counter-defense mechanism against viral suppressors of RNA interference (VSRs). The RNA interference (RNAi) pathway serves as a primary antiviral response in insects, but viruses, such as Flock House virus (FHV), have evolved VSRs like B2 to disrupt this defense. Our research identifies cyclin-dependent kinase 12 (CDK12) as a critical host factor that interacts with the VSR B2. The discovery that CDK12 can counteract B2-mediated RNAi suppression and stimulate the production of viral small interfering RNAs (vsiRNAs) in FHV-infected *Drosophila* cells highlights its pivotal role in enhancing antiviral RNAi immunity. This study not only reveals a new dimension of host-virus interactions but also opens avenues for developing strategies to strengthen RNAi-based antiviral defenses.

## INTRODUCTION

*Drosophila melanogaster* has been a powerful model for studying antiviral innate immunity, largely due to its lack of adaptive immune response and the fact that evolutionary conservation of its innate immunity with vertebrates. In *Drosophila*, the primary antiviral defense mechanism is the conserved RNAi pathways, which are also present in plants, fungi, invertebrates, and mammals. The initiation of antiviral RNAi depends on the generation of viral-derived double-stranded RNA (dsRNA), derived from either replicative intermediate generated during RNA virus replication or complementary transcripts of DNA viruses (1). DsRNAs are recognized by the host endoribonuclease Dcr-2 and cleaved into 21- nucleotide (nt) virus-derived siRNAs (vsiRNAs) with two unpaired nucleotides at the 3' end of either strand (2). Subsequently, Dcr-2 transports these vsiRNAs to AGO2, where they are incorporated into the RNA interference silencing complex (RISC). This complex guides the cleavage of viral RNAs that exhibit perfect sequence complementarity to vsiRNAs, thereby impeding viral replication (3, 4).

To counteract antiviral RNAi immunity, viruses have developed viral suppressors of RNAi (VSR) (5, 6). These VSRs are crucial for efficient viral replication within the host and have independently evolved with diverse mechanisms to suppress the antiviral RNAi pathway (7). Notably, various VSRs employ distinct strategies to impede the host’s antiviral defenses. For example, proteins 1A and VP3, encoded by *Drosophila* C virus and *Drosophila* X virus, respectively, bind to dsRNA to prevent Dcr-2-mediated cleavage of viral dsRNA (8–10). In contrast, protein 1A of the Cricket Paralysis virus directly binds to AGO2, inhibiting the cleavage of viral RNA (11–13). In addition, B2, expressed by subgenome 3 of Flock House virus (FHV), is a well-studied VSR composed of only 106 amino acids and three α-helices (14, 15). B2 employs multiple mechanisms to suppress the antiviral RNAi pathway, including the following: (i) dimerization that binds to dsRNAs and siRNAs, inhibiting the production of vsiRNAs and suppressing the assembly of vsiRNAs into RISCs, respectively (15–18), and (ii) direct binding to Dcr-2, inhibiting its cleavage activity (16, 19).

The antagonistic co-evolution between viruses and hosts has led to the existence of a counter-defense mechanism in the host that responds to the defensive effects of VSR, which has been termed a counter counter-defense strategy (20). In our recent findings, we discovered that VINR, a *Drosophila* long non-coding RNA (lncRNA), triggers a non-classical innate immune response against the VSR of *Drosophila* C virus (21). However, the understanding of host defenses against VSRs in *Drosophila* remains limited, and the host factors involved in counteracting RNAi suppression by VSR need to be identified.

Cyclin-dependent kinase 12 (CDK12) is a serine/threonine protein kinase with evolutionarily conserved features. In *Drosophila,* CDK12 shares homology with the pair of isozymes CDK12/CDK13 in humans, particularly in the sequence of the catalytic kinase domain, which exhibits a high degree of conservation (22). The most well-characterized role of CDK12 is to phosphorylate threonine residues in the C-terminal repeat domain of RNA polymerase II, which regulates the initiation and elongation of mRNA transcription, mRNA splicing, and polyadenylation (22–24). Interestingly, CDK12 does not regulate global gene expression like other CDK families but involved in cellular stress, heat shock, and DNA damage response (25, 26). Additionally, CDK12 serves as an antagonist to heterochromatin enrichment in *Drosophila* chromosomes and contributes to the maintenance of transcription for neuronal genes linked to learning (27). Furthermore, recent studies have highlighted the critical role of CDK12 in human cancer development (28–31). Dubbury et al. reported that CDK12 represses tumorigenesis by suppressing intronic polyadenylation and regulating the expression of homologous recombinant genes (32). In addition, CDK12 has been proven to regulate cellular signaling pathways and immune pathways associated with tumorigenesis and progression, including the c-MYC/β-catenin signaling pathway, ErbB–PI3K–AKT pathway, WNT signaling cascade, MAPK signaling pathway, noncanonical NF-κB pathway, and DNA damage response signaling pathway. However, little is known about the potential function of CDK12 in antiviral innate immunity.

In this study, we aim to identify the host factors in counteracting RNAi suppression by VSR and elucidate detailed mechanisms. We identified that CDK12 interacted with the VSR encoded by FHV, named B2, and that only in the context of B2, CDK12 inhibited viral replication and the production of vsiRNAs, the effectors of the antiviral RNAi pathway. This discovery highlights a unique strategy employed by CDK12 to counteract the viral suppression of the primary antiviral RNAi immunity in *Drosophila melanogaster*, shedding light on a sophisticated counter-defense mechanism.

## RESULTS

### Identification of FHV B2-associated host proteins by LC-MS/MS

**Fig 1:**
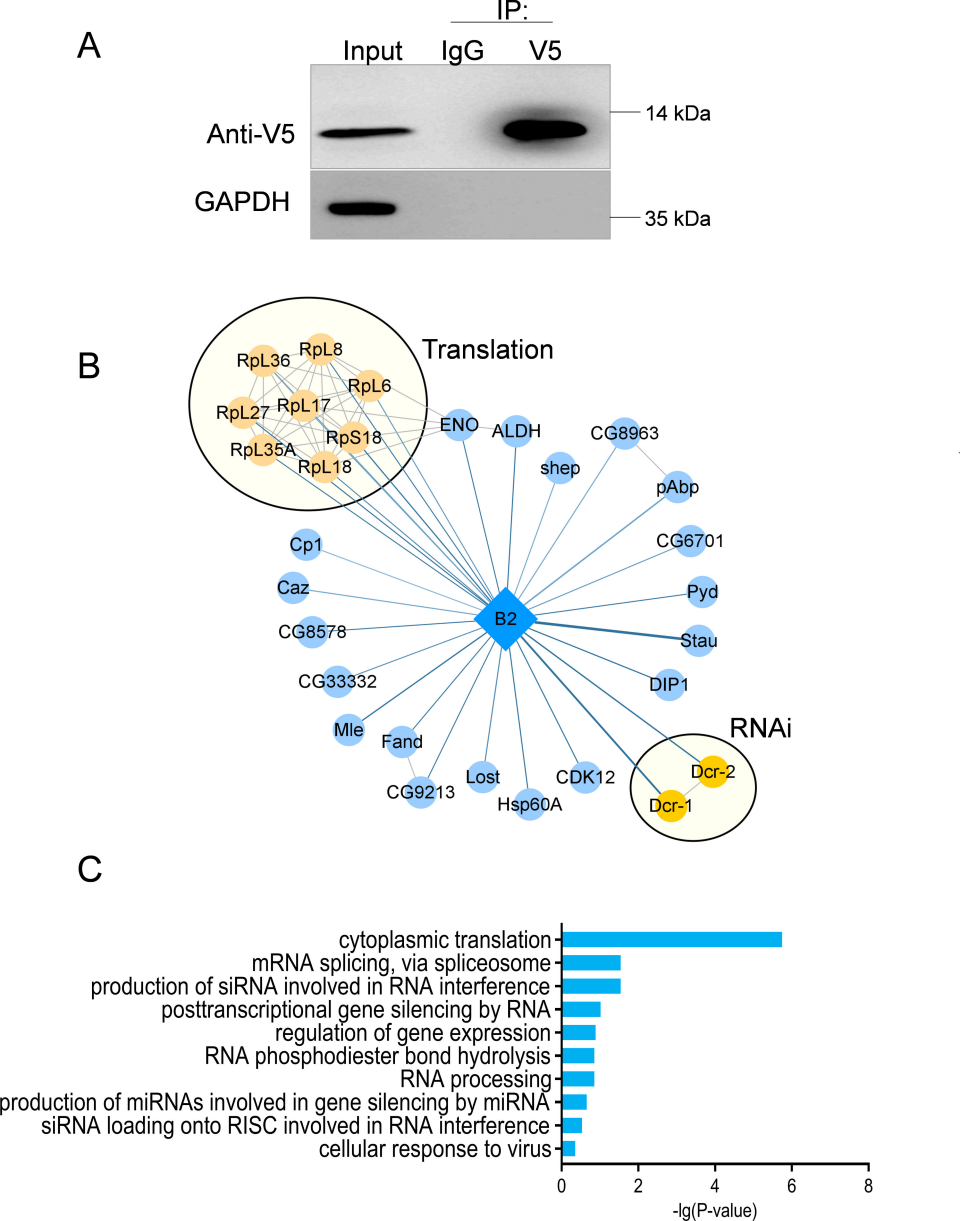
Bioinformatic analysis of interacting proteins of FHV B2. (**A**) S2 cells transfected with the V5-tagged B2 plasmid for 48 h were subjected to Co-IP using anti-V5 or anti-IgG antibodies and western blotting for immunoprecipitates using the indicated antibodies. (**B**) The interactions between B2 and cellular host proteins (diamond represented B2; circles represented cellular host proteins; yellow indicated proteins in the same biological process). (**C**) Functional enrichment analysis for the B2 interacting proteins identified by LC-MS/MS of immunoprecipitates using an anti-V5 antibody, compared with anti-IgG antibody.

To explore and identify potential host proteins associated with the B2 protein, a VSR of FHV, we transfected a V5-tagged B2 expression vector or an empty vector control into Schneider2 (S2) cells and then immunoprecipitated the cell lysates with anti-V5 antibody (Fig. 1A). Subsequently, proteins that coprecipitated with B2 were analyzed by liquid chromatography-tandem mass spectrometry (LC-MS/MS). Our LC-MS/MS analysis identified 135 B2-interacting proteins, as indicated by unique peptides with more than two occurrences (Table S1). We applied MiST scoring algorithms to calculate the statistical significance. Utilizing four cutoffs, namely unique peptide count > 2, MS score > 10, fold change > 2, and Mist score > 0.8, we narrowed down our results to 29 B2-interacting proteins.

To construct PPI network, uniProt IDs were converted to *Drosophila melanogaster* gene IDs, and interaction networks were generated in StringDB with a minimum required interaction score of 0.9. The analysis resulted in 65 high-confidence interactions and mainly results from a number of ribosomal proteins clustered within the network (Fig. 1B). Notably, B2 was shown to interact with Dcr-2, consistent with previous findings that B2 suppresses RNA silencing by directly interacting with Dcr-2 in *Drosophila* cells (19, 33). Interestingly, we also identified Dcr-1, known for its role in miRNA biogenesis. Although some functional overlap exists with Dcr-2, a detailed analysis of the interaction between B2 and Dcr-1 requires further investigation. Moreover, our study revealed numerous RNA processing proteins, including RNA binding proteins Stau, CG8963, CG6701, pAbp, shep, and DIP1, of which DIP1 has been reported to play an antiviral role in *Drosophila* (34). Additionally, RNA splicing proteins Fand, CG9213, and enzymes associated with RNA processing, such as CDK12, ALDH, ENO, and CG8578 were identified (Fig. 1B). Furthermore, Gene Ontology (GO) enrichment analysis revealed that the host cellular pathways were involved in cytoplasmic translation, mRNA splicing, and RNA interference (RNAi) pathways (Fig. 1C), suggesting that those B2-interacting proteins play a role in the RNAi pathway.

### CDK12 restricts FHV replication but not B2-deficient virus

**Fig 2:**
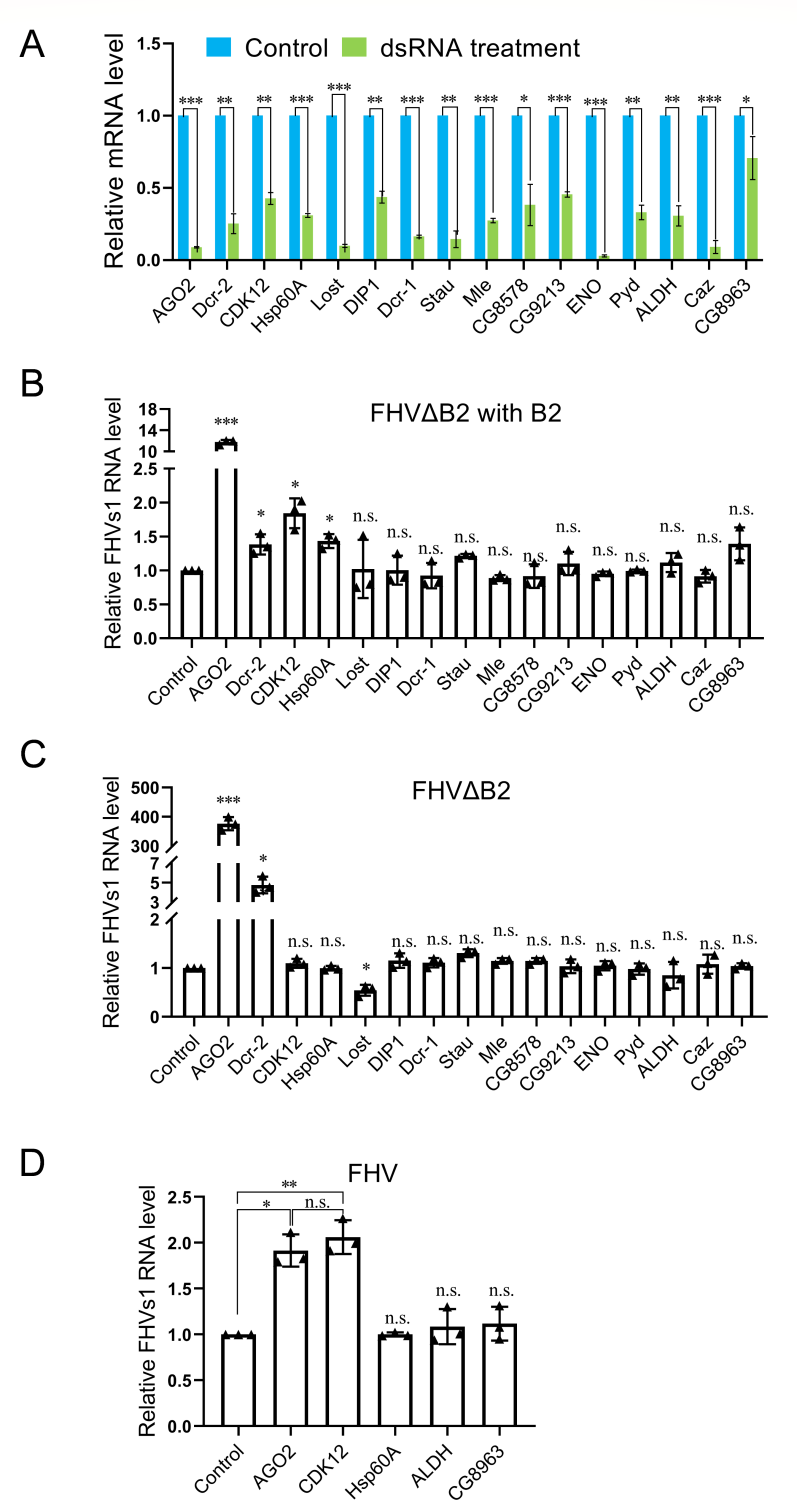
CDK12 restricts FHV replication but not FHVΔB2. (**A**) RT‒qPCR analysis of the mRNA levels in S2 cells treated with the indicated dsRNAs. β*-gal* dsRNA was used in the control. (**B and C**) S2 cells were pretreated with dsRNA against a control (β*-gal*) or the indicated genes for 48 h and then transfected with pMT-B2-V5 (**B**) or pMT-GFP (**C**) plasmids for another 48 h. The pretreated cells were infected with FHVΔB2 and the FHV RNA1 levels, relative to the control, were determined by RT‒qPCR at 24 hpi. (**D**) S2 cells were pretreated with dsRNA against a control (β*-gal*) or the indicated genes for 48 h and infected with FHV (MOI = 1), prior to viral RNA quantification with RT‒qPCR at 24 hpi. The relative mRNA or viral RNA levels were normalized to the controls (**A–D**). Error bars represent SD in triplicate experiments (**A–D**). Statistical analysis was performed for panels **A–D**; ∗*P* < 0.05, ∗∗*P* < 0.01, ∗∗∗*P* < 0.001, n.s., not significant (Student’s *t*-test).

To further investigate the potential impact of these identified proteins on the function of B2 in FHV infection, we initially tested the first 17 candidate proteins, with AGO2 protein as a positive control. Each gene was effectively knocked down by treatment with dsRNA (Fig. 2A). Remarkably, the numbers of cells treated with dsRNA against fand and pAbp genes were significantly reduced (data not shown), suggesting that these two genes are crucial for cell survival. Subsequently, the knockdown cells were then transfected with Flag-tagged B2 plasmids or control plasmids before being infected with a B2 deficient virus (FHVΔB2). Knockdown of AGO2 and Dcr-2 in the absence of B2 protein dramatically increased FHV genome RNA levels by 376-fold and 4.8-fold, respectively (Fig. 2C). In the presence of B2 protein, FHV genome RNA levels remained elevated, but the fold changes decreased to 11.8-fold and 1.4-fold, respectively (Fig. 2B). These results align with the known function of B2 as a VSR. Intriguingly, in the presence of B2, CDK12 and Hsp60A knockdown significantly increased FHV replication, whereas these effects disappeared in the absence of B2 protein (Fig. 2B and C), suggesting that CDK12 and Hsp60A inhibit the protective effect of B2 on FHV replication.

To further validate the impact of CDK12 and Hsp60A on wild-type FHV, we conducted knockdown experiments in S2 cells, utilizing Aldh and CG8963 as negative controls. Following knockdown, cells were infected with FHV. We observed that the silencing of CDK12 significantly increased the FHV viral RNA levels in S2 cells, and this increase was slightly higher than in cells with AGO2 silencing, although no significance was observed (Fig. 2D). In contrast, Hsp60A, Aldh, and CG8963 exhibited no discernible effect on FHV replication (Fig. 2D). Taken together, these findings strongly suggest that the loss of CDK12 enhances the susceptibility of S2 cells to FHV, a phenomenon not observed in the context of FHVΔB2.

### CDK12 modulates FHV replication and RNAi in the presence of B2

**Fig 3:**
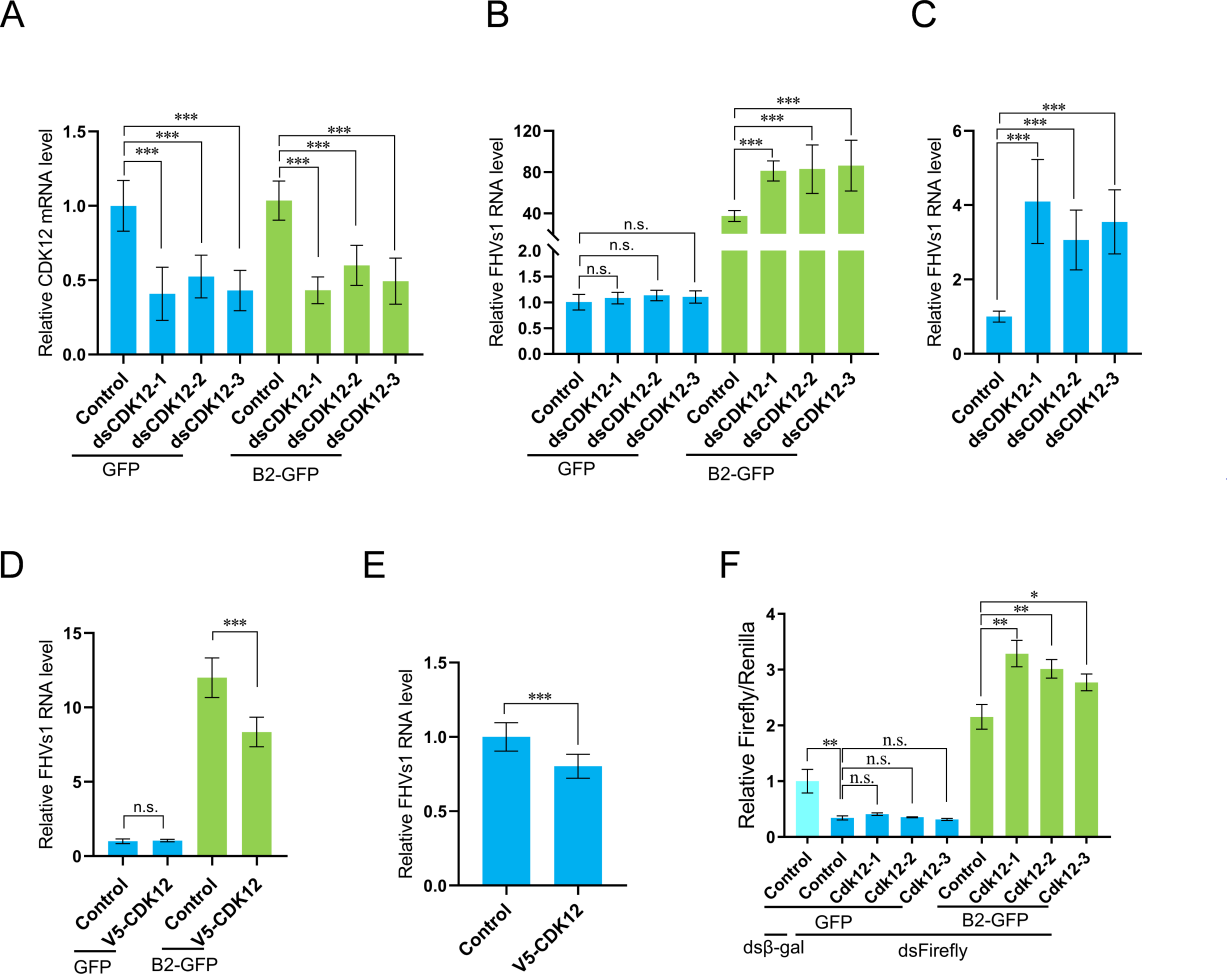
CDK12 induced antiviral RNAi response by inhibiting the function of B2. (**A and B**) S2 cells were pretreated with three different dsRNAs targeting CDK12 or control dsRNA (β*-gal*) for 48 h and transfected with pMT-B2-GFP or pMT-GFP plasmids for another 48 h. The GFP-positive cells were sorted by FACS and followed by FHV∆B2 infection. The total RNA was isolated at 24 hpi. The CDK12 mRNA levels (**A**) and FHV RNA1 levels (**B**), relative to the control in GFP group, were determined by RT‒qPCR. (**C**) S2 cells were pretreated with three different dsRNAs targeting CDK12 or control dsRNA (β*-gal*) for 48 h and infected with FHV (MOI = 1), prior to viral RNA quantification with RT-qPCR at 24 hpi. (**D**) S2 cells were co-transfected with pMT-B2-GFP and pMT-GFP, pMT-B2-GFP and pMT-V5-CDK12, pMT-GFP and pMT-V5-CDK12, or pMT-GFP for 48 h and infected with FHVΔB2, prior to viral RNA quantification with RT‒qPCR at 24 hpi. (**E**) S2 cells transfected with pMT-V5-CDK12 and pMT-GFP for 48 h and infected with FHV (MOI = 1), prior to FHV RNA quantification with RT-qPCR at 24 hpi. (**F**) S2 cells were pretreated with three different dsRNAs targeting CDK12 or control dsRNA (β*-gal*) for 48 h and then co-transfected with psi-CHECK2 plasmids and pMT-B2-GFP or pMT-GFP plasmids for another 48 h. The pretreated cells were treated with dsRNAs against firefly luciferase or β*-gal*, and both firefly and Renilla luciferase activity were measured at 48 h. The activity ratios between firefly luciferase and Renilla luciferase were shown and normalized to the control in dsRNA targeting β*-gal* group. Error bars represent SD in three independent experiments performed in triplicate. Statistical analysis was performed using ANOVA; ∗∗∗*P* < 0.001, n.s., not significant.

To further confirm the impact of CDK12 on FHV replication, we designed two additional dsRNAs targeting distinct regions of CDK12 mRNA to exclude potential off-target effects. Employing RNAi, we successfully achieved the knockdown of CDK12 using these three dsRNAs in S2 cells, as illustrated in Fig. 3A. In alignment with the findings presented in Fig. 2B and C, all three dsRNA treatments significantly enhanced FHV replication specifically in the presence of B2 while exhibiting no discernible effects in the absence of B2 (Fig. 3B). Previous studies have demonstrated that the functions of the DCV VSR protein 1A and FHV B2 are interchangeable (17, 21). In our study, we confirmed that DCV-1A can rescue FHV-ΔB2 replication to levels comparable with the B2 protein under CDK12 knockdown conditions (Fig. S1A). Furthermore, the accumulation of wild-type FHV RNAs was significantly elevated in all three CDK12-knockdown cells (Fig. 3C). Notably, the overexpression of CDK12 markedly inhibited FHVΔB2 replication in S2 cells co-transfected with B2 plasmids; however, this inhibitory effect was entirely abolished in cells lacking co-expressed B2 (Fig. 3D). Moreover, the overexpression of CDK12 significantly enhanced suppression of wild-type FHV replication (Fig. 3E). These data underscore that CDK12 exerts an antiviral effect exclusively in the context of B2. Given the known functions of the B2 protein in RNAi pathway, we next explored the potential involvement of CDK12 in this pathway by using a dual luciferase reporter system. We observed no significant differences in the RNAi targeting the *firefly* luciferase gene between the wild-type and CDK12 knockdown cells (Fig. 3F), suggesting that CDK12 is non-essential for RNAi activation. In contrast, the expression of B2 dramatically increased the level of *firefly* luciferase (Fig. 3F), confirming that B2 effectively inhibited the RNAi response in S2 cells. Furthermore, upon comparing the expression of *firefly* luciferase in wild-type and CDK12 knockdown cells in the presence of B2, we found that *firefly* luciferase activities were significantly increased in all three CDK12 knockdown cells (Fig. 3F), indicating that the inhibitory effects of B2 protein on the RNAi response were more potent in CDK12 knockdown cells. Overall, these data revealed that CDK12 maintains the RNAi response by inhibiting the VSR function of B2 protein in S2 cells.

### Key sites for dimerization and dsRNA binding of B2 required for interaction with CDK12

**Fig 4:**
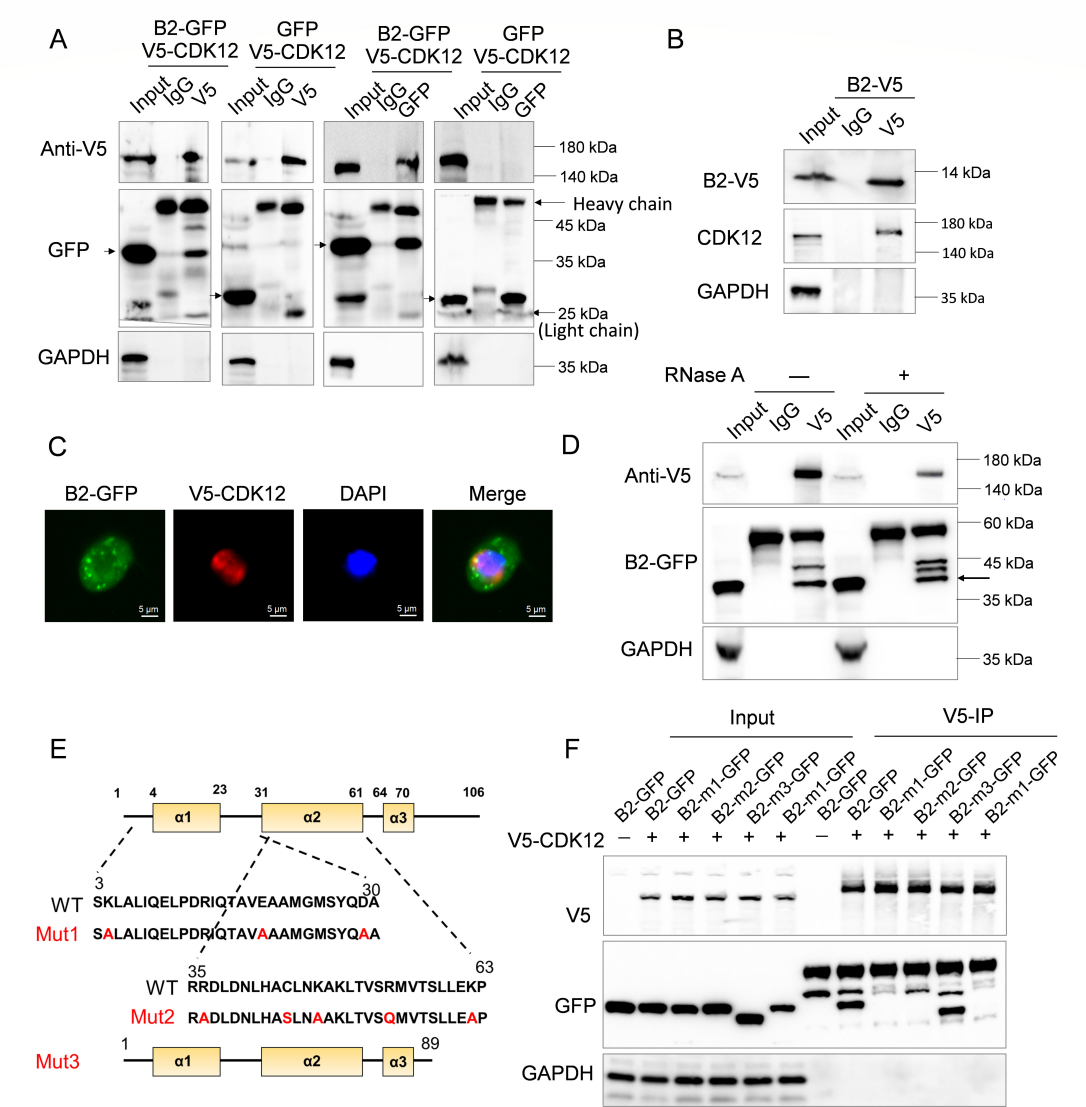
The critical amino acids responsible for dsRNA binding and dimerization in B2 were essential for its interaction with CDK12. (**A**) S2 cells co-transfected with pMT-V5-CDK12 plasmid and pMT-B2-GFP or pMT-GFP plasmids were subjected to Co-IP using anti-V5 (left) or anti-GFP (right) and western blot for the immunoprecipitates using the indicated antibodies. Black arrows indicate the target proteins. (**B**) S2 cells transfected with pMT-B2-V5 plasmid for 48 h and infected with FHVΔB2, prior to Co-IP using anti-V5 antibodies, and western blot for the precipitates using anti-CDK12, anti-V5, and anti-GAPDH antibodies. (**C**) Representative images from immunofluorescence staining of co-localization between B2 and CDK12 in S2 cells co-transfected with pMT-V5-CDK12 and pMT-B2-GFP plasmids. (**D**) Lysates from S2 cells co-expressing V5 tagged CDK12 and GFP-tag B2 were untreated or pretreated with RNase A, prior to Co-IP using anti-V5 antibodies. Western blot for the immunoprecipitates using the indicated antibodies. Black arrows indicate the target proteins. (**E**) Schematic representation of B2 containing three α-helices and three mutants deficient in key amino acids for dimerization, dsRNA binding, or Dcr-2 binding functions of B2, respectively. (**F**) S2 cells were co-transfected with pMT-V5-CDK12 plasmid and pMT-B2-GFP or GFP-tagged B2 mutants expressing plasmids, and cells without V5-CDK12 expression were used as a negative control, prior to Co-IP using anti-V5 antibody. The immunoprecipitates were subjected to western blotting using the indicated antibodies.

To investigate the interaction between B2 and CDK12 in S2 cells, we conducted co-transfection experiments with V5-CDK12 and either B2-GFP or GFP-expressing plasmids. Subsequently co-immunoprecipitation was performed with a V5 antibody. Our results clearly indicated the interaction between B2 and CDK12, whereas GFP alone could not be co-precipitated by V5-CDK12 (Fig. 4A). Furthermore, V5-CDK12 was detected in the immunoprecipitants of B2 (Fig. 4A). Consistent with this, we confirmed that B2 interacts with endogenous CDK12 (Fig. 4B). Immunofluorescence staining further revealed that CDK12 and B2 predominantly co-localize in the cytoplasm of S2 cells (Fig. 4C). Given that B2 and CDK12 are both RNA-binding proteins, we further tested whether the interaction between CDK12 and B2 was mediated by RNA. Cell lysates were treated with and without RNase A before performing co-immunoprecipitation, and the results showed that RNase A treatment did not alter the interaction between B2 and CDK12 (Fig. 4D). In addition, previous study has shown that the functionality of DCV-1A and B2 is interchangeable (17, 21). We confirmed that DCV-1A rescues the replication defect of the FHV-ΔB2 replicon; however, unlike FHV B2, DCV-1A does not interact with CDK12 (Fig. S1A and B), implying that CDK12 might direct affect B2 functions. Thus, our investigation focused on delineating the pivotal CDK12-binding site of B2. To achieve this, three plasmids expressing B2 mutants were constructed. As illustrated in Fig. 4E, mutant 1 harbored mutations in key dimerization sites (K4A, E20A, and D29A) (16), mutant 2 featured mutations in critical dsRNA recognition sites (C44, R36, K47, R5, and K62) (15, 17, 18), and mutant 3, lacking the C-terminal 17 amino acids, demonstrated an inability to interact with Dcr-2 (ΔC17) (19). Our results revealed that mutant 3 exhibited CDK12-binding comparable with wild-type B2. In contrast, mutants 1 and 2 were undetectable in the immunoprecipitants using the V5 antibody, akin to the negative control not co-transfected with the V5-CDK12 plasmid (Fig. 4F). Collectively, these findings underscore the indispensability of amino acids responsible for dimerization and dsRNA recognition in facilitating CDK12 binding.

### CDK12 is required for vsiRNAs production in FHV-infected cells

**Fig 5:**
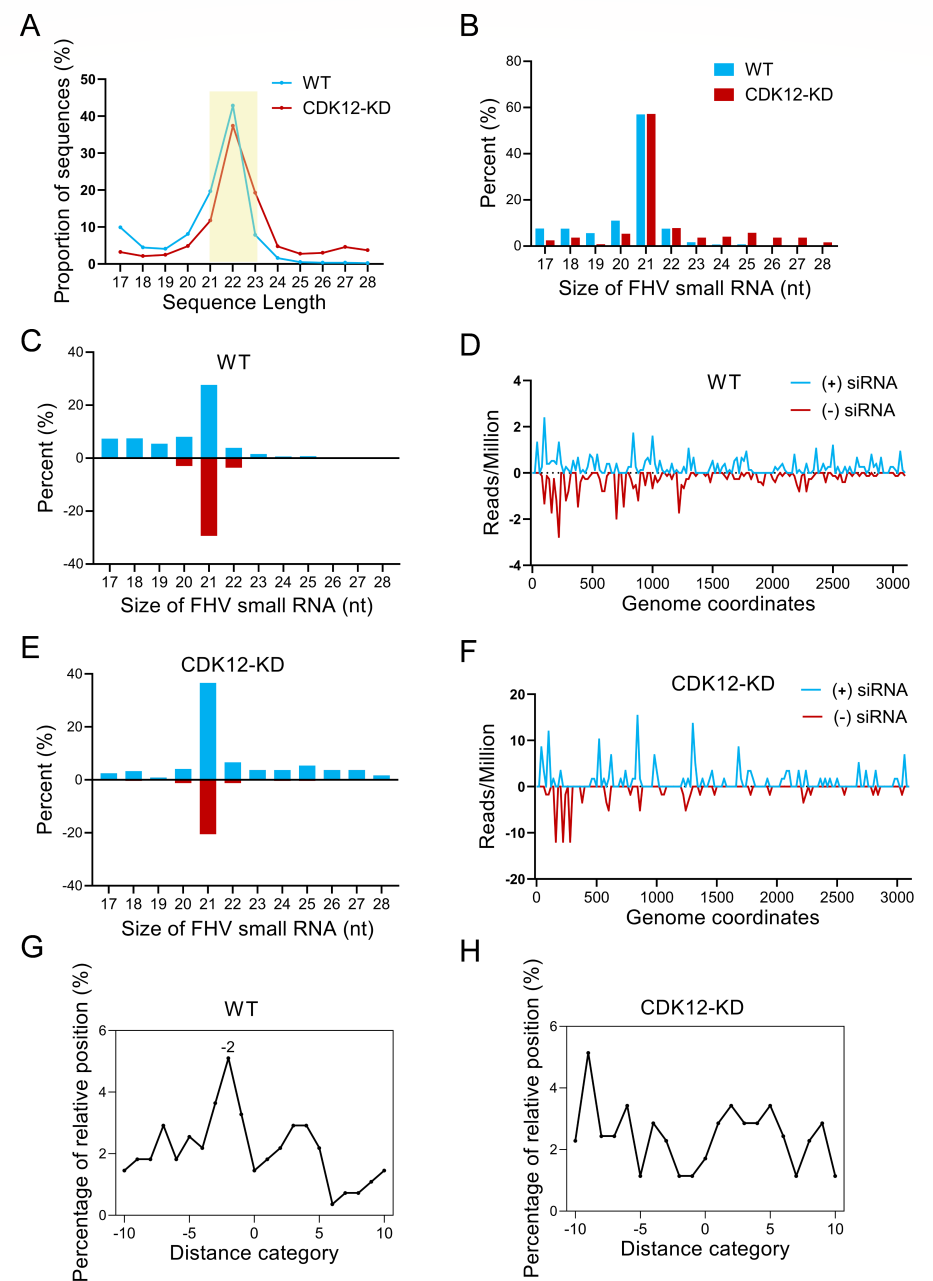
CDK12 is required for FHV-derived siRNA production. (**A and B**) Length distributions of total small RNAs (**A**) or FHV-derived small RNAs (**B**) sequenced from wild-type or CDK12 knockdown cells infected with FHV (MOI = 1) at 24 hpi. The yellow shade indicated small RNA peaks. (**C and E**) Distributions of each size of FHV-derived small RNA aligned to the positive and negative strands of the FHV genome in wild-type cells (**C**) or CDK12 knockdown cells (**E**). blue, positive-stranded vsiRNAs; red, negative-stranded vsiRNAs. (**D and F**) The distribution of 21 nt-long vsiRNAs reads in the positive and negative-stranded FHV RNA1 and the relative abundances of positive and negative-stranded vsiRNAs (counts per million, RPM) are indicated in wild-type cells (**D**) or CDK12 knockdown cells (**F**). (**G and H**) Total pairs of complementary 20 to 22-nt vsiRNAs derived from wild-type cells (**G**) or CDK12 knockdown cells (**H**) in each distance category between 5′ and 3′ ends of a complementary vsiRNA pair, shown as −two for pairs with 2-nt overhang at the 3′ end of each strand defined as the canonical vsiRNAs.

We next aimed to investigate whether CDK12 targets and inhibits B2 to unlock the antiviral activity of RNAi. Previous studies have highlighted the integral role of B2 in inhibiting vsiRNAs production (14). To further validate the necessity of CDK12 in vsiRNAs production, small RNA libraries were prepared from FHV-infected CDK12 knockdown and wild-type S2 cells, followed by deep sequencing using the Illumina HiSeq 2500 platform. After adapter trimming, 17–28 nt reads were used for subsequent analysis. We observed that the length distributions were comparable between the two conditions, predominantly falling within the 21–23 nt range, with 70.4% and 68.4% of total reads in wild-type and CDK12 knockdown cells, respectively. Notably, the 22 nt small RNA peaks, reflecting the dominant size of miRNAs, accounted for 42.8% and 37.4% of the total reads in wild-type and CDK12 knockdown cells, respectively (Fig. 5A).

**Fig 6:**
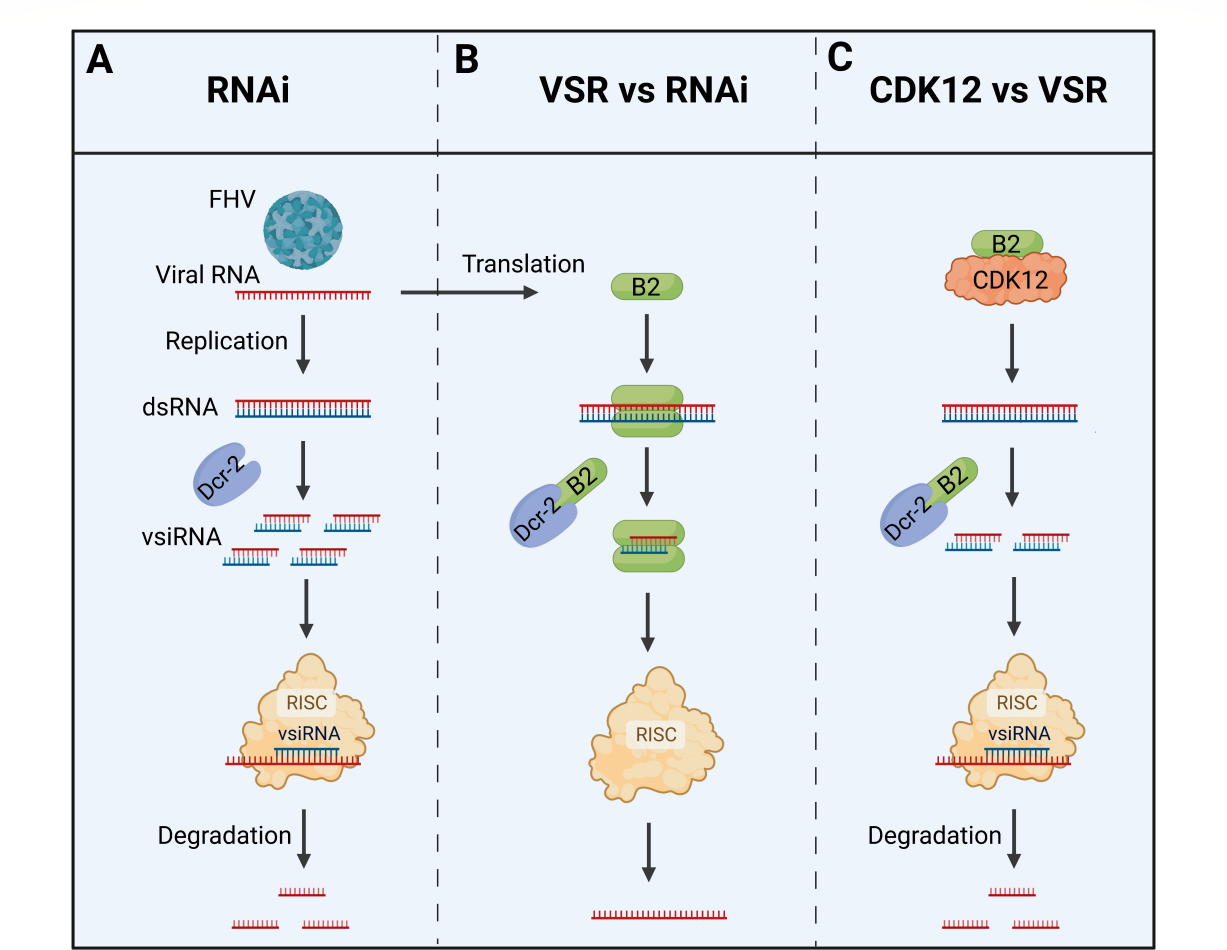
The proposed model of CDK12 against VSR to maintain host antiviral RNAi immunity. (**A**) In *Drosophila* cells, dsRNA generated during FHV replication induces host antiviral RNAi immunity, which produces the antiviral effector, vsiRNAs, that results in the degradation of viral RNA and suppression of viral replication. (**B**) FHV encodes B2 protein to counteract antiviral RNAi immunity in multiple ways, including by binding to long dsRNA, interacting with Dcr-2, and binding to siRNA, and B2 is a viral virulence factor necessary for FHV replication. (**C**) CDK12 inhibits the VSR function of B2 and enhances vsiRNAs production, resulting in more potent RNAi immunity against viral infection.

Considering only small RNAs exhibiting 100% identity or complementarity to the bipartite RNA genome of FHV (GenBank accession no. NC_004146 and NC_004144) as viral-derived small RNAs (vsiRNAs), we observed major peaks of vsiRNAs were at 21 nt, accounting for 57.0% and 57.2% of the total mapped to FHV in wild-type and CDK12 knockdown cells, respectively (Fig. 5B). The length distribution of vsiRNAs from FHV aligned with previous reports (14). Intriguingly, the distribution of mapped vsiRNAs across the FHV genome differed between wild-type and CDK12 knockdown cells. In the wild-type library, 21 nt-long vsiRNAs were evenly distributed between positive and negative strands of the FHV genome (48.3% positive strand-derived siRNAs and 51.7% negative strand-derived siRNAs) (Fig. 5C). Additionally, positive strand-derived vsiRNAs largely mapped to the same genomic positions as those derived from negative strands (Fig. 5D), consistent with the notion that vsiRNAs are double-stranded structures produced by Dcr-2 processing. However, the 21 nt-long vsiRNAs from CDK12 knockdown cells displayed a higher percentage for positive strands (64% of positive strand-derived siRNAs and 36% of negative strand-derived siRNAs) (Fig. 5E). Additionally, positive strand-derived vsiRNAs mapped to distinct genomic positions compared with negative strand-derived vsiRNAs (Fig. 5F), suggesting that they are unlikely to be Dcr-2 processed siRNA duplexes. These results imply that some of the vsiRNAs were likely breakdown products from the abundant positive-strand viral RNA in CDK12 knockdown cells. More importantly, FHV-derived siRNA exhibited strong enrichment for canonical siRNA duplexes, characterized by a 19-nt perfectly base-paired duplex region with 2-nt 3' overhangs (Fig. 5G), although this characteristic was not observed in CDK12 knockdown cells (Fig. 5H). Together, these findings suggested that CDK12 enhanced the process of viral dsRNAs into siRNAs during FHV infection (Fig. 6).

## DISCUSSION

RNAi has been well recognized as an intrinsic antiviral defense mechanism in both plants and animals (1). As a counter-defense strategy, many viruses encode VSR to evade the antiviral RNAi pathway. Conversely, certain host factors have been identified to counteract VSR activity in plants (35–37), whereas no host factors antagonizing VSRs in animals have been reported. To the best of our knowledge, this study presented the first identification of host potential interaction proteins for FHV B2 in S2 cells. We systematically tested 17 proteins with higher confidence, aiming to identify those that could influence viral VSRs and RNAi pathways in FHV replication. Our findings reveal that the host CDK12 protein interacts with B2 and the key amino acids responsible for dimerization and dsRNA binding in B2 are indispensable for CDK12 binding. This interaction leads to the suppression of VSR functions and further influences RNAi antiviral activities, highlighting a counter-counter-defense strategy in *Drosophila* (Fig. 6).

CDK12, a cyclin-dependent kinase, plays essential roles in DNA replication, transcription, mRNA splicing, and DNA damage repair. Although its functions have been extensively studied in cancer (28–32), the role of CDK12 in viral infection has not been previously reported. In this study, we demonstrate that CDK12 interacts with the viral B2 protein and counteracts its inhibitory effect on the RNAi pathway. The human CDK family consists of over 20 members, with their emerging roles in antiviral innate immunity recently reviewed (38, 39). For instance, CDK12 and CDK13 share the highest sequence homology among CDKs and previous studies have shown that the HIV-1 Tat protein interacts with CDK13 *in vivo* and *in vitro* (40). Additionally, in our study, we observed that both CDK12 and the viral B2 protein primarily co-localize in the cytoplasm. Consistent with this, prior studies have shown that CDK12 localizes to both the cytoplasm and the nucleus, with its translocation potentially facilitated by cyclin K (41, 42). Our findings introduce a new role for CDK12, highlighting its involvement in antiviral defense within the cytoplasm, beyond its established function in regulating nuclear gene expression. Interestingly, we observed that the FHV replication in cells with CDK12 silenced was slightly higher than that in cells with AGO2 silenced (Fig. 2D). The well-established role of CDK12 is to regulate gene expression, especially for genes involved in processes such as heat shock and cellular stress response (25, 26). It has been reported that many heat shock proteins, such as HSP70, HSP40, and HSP90, play critical roles in the infection of FHV (43–45). This indicated that CDK12 may play a role in host antiviral defense by regulating gene expression.

FHV B2 employs multiple mechanisms to inhibit RNAi pathway in the cytoplasm. First, B2 binds to dsRNA and siRNAs, inhibiting the siRNA pathway by sequestering siRNAs and preventing the processing of dsRNA (15–18). Second, B2 directly interacts with Dcr-2 and inhibits its activity (16, 19). Previous studies have demonstrated that the FHV B2 protein forms a dimeric structure to bind dsRNA, with a pair of antiparallel arrangements of helices α2 forming a binding surface with dsRNA, and mutation of the charged amino acids on helix α2 results in a loss of the ability of B2 to bind dsRNA (15, 18). In the absence of dsRNA, B2 can also form dimers, with helix α1 playing an important role in the stability of the dimer (15, 16). Our results reveal that the key charged amino acids for B2 dimerization and dsRNA binding were essential for CDK12 binding (Fig. 4C). This suggests that CDK12 may prevent B2-dsRNA binding through affecting the conformation or dimerization of B2, thereby inducing the production of vsiRNAs that mediate the degradation of viral RNAs in FHV-infected cells. Further studies are necessary to elucidate the precise mechanism of how CDK12 inhibits B2 to unlock the antiviral activity of RNAi.

We compared the characterization of FHV-derived siRNAs in FHV-infected wild-type and CDK12 knockdown cells. In FHV-infected wild-type cells, a major peak of vsiRNAs was at 21 nt, and the numbers of 21 nt-long vsiRNAs derived from the positive and negative strands were equivalent (Fig. 5B and C). Our results are consistent with previous studies showing that the amount of positive-strand RNA was comparable with that of negative-strand RNA during the early stages of viral infection (46). However, some controversial findings have reported that positive-strand-derived vsiRNAs were more abundant (47, 48). A possible explanation for these discrepancies could be differences in experimental conditions, such as virus multiplicity and infection duration. In certain cases, the positive-strand RNA of the FHV genome may not have significantly increased, leading to differences in the vsiRNA profile. The 5‘-terminal region of FHV RNA1 was covered with more abundant vsiRNAs (Fig. 5D), consistent with previous results (14). Moreover, vsiRNAs displayed a significant 2-nt overhang at the 3’ end in wild-type cells (Fig. 5G), suggesting that the RNAi response exerts an antiviral effect in FHV-infected cells. However, we found that 21 nt-long vsiRNAs displayed an overwhelming bias for positive strands and lacked a 2-nt 3‘overhang in CDK12 knockdown cells (Fig. 5E and F), indicating that CDK12 inhibited the replication of FHV by inducing vsiRNAs production. In addition, we found that B2 mutant 3, which lacked Dcr-2 binding domain, bound to CKD12 at the same level as that of wild-type B2, illustrating that the binding site of B2 to CDK12 is different from that to Dcr-2 and may imply that CDK12 does not affect the inhibition of Dcr-2 activity by B2 (Fig. 4F). However, whether CDK12 affects vsiRNAs being assembled into RISC by preventing B2-siRNA binding remains to be investigated.

Overall, we employed FHV infection in the *Drosophila* S2 cell line as a model and identified CDK12 as an additional layer of defense. This discovery reveals that the host has the capability to antagonize the VSRs mediated by the FHV B2 protein, indicating the existence of a robust host counter-counter-defense mechanism in *Drosophila*. These findings contribute to our understanding of the ongoing arms race between viruses and their hosts, shedding light on the intricate dynamics of host-pathogen interactions.

## MATERIALS AND METHODS

### Plasmids construction

The cDNA of FHV B2 was amplified by RT-PCR from the FHV genome (GenBank accession no. NC_004146). The sequences of the GFP tag were fused to the C-terminal of B2 by overlapping PCR, and the PCR products were cloned into pMT/V5-His A vector. B2-mutant1-GFP and B2-mutant2-GFP fragments were generated by overlapping PCR with primers containing the indicated mutated bases, and the B2-mutant3-GFP was generated by PCR with primers at the truncated site. These fragments were inserted into the pMT/V5-His A vector.

The CDS of CDK12 were amplified by RT-PCR from the total RNA of S2 cells, and the sequence of V5 tag was fused to N-terminal of CDK12 by overlapping PCR. The PCR product was cloned into pMT/V5-His A vector. All plasmids were confirmed using Sanger sequencing (Tsingke, China). The primers for all expression plasmids are listed in Table S2.

### Virus preparation

FHV virions were purified following a published method with modifications (49). Briefly, S2 cells were inoculated with FHV and were harvested at 3 days post-infection. The cells were lysed with buffer A (50 mM HEPES [pH 7.0], 5 mM CaCl_2_, 0.5% NP40, and 0.1% β-mercaptoethanol) and subjected to three freeze/thaw cycles. After centrifugating at 14,000 rpm at 4°C for 30 min, the supernatant was incubated with 10 µg of RNase A/mL for 30 min at 27°C. The reaction mixture was subjected to centrifugation at 14,000 rpm at 4°C for 30 min. The supernatant was overlaid on a 30% sucrose cushion in buffer B (50 mM HEPES [pH 7.0], 5 mM CaCl_2_, 0.1% bovine serum albumin, and 0.1% β-mercaptoethanol) and ultracentrifugated at 25,000 rpm for 3 h. The virion pellet was resuspended in HEPES buffer, and the insoluble substance was removed by centrifugation at 14,000 rpm at 4°C for 30 min. Virus stock was titrated by end-point dilution, and S2 cells were inoculated with a dilution of virus. Cytopathic effect was monitored visually over 14 days. Titer was calculated as TCID50/mL according to the Reed-Muench method. The FHV∆B2 mutant strain was kindly provided by Prof. Qingfa Wu (University of Science and Technology of China, Hefei, China) and was propagated as previously described (14). Briefly, the S2 cells were first subjected to RNAi-mediated knockdown of AGO2, followed by infection with FHV∆B2. Infected cells were harvested 48 h post-infection, subjected to three freeze-thaw cycles, and centrifuged at 14,000 rpm for 30 min at 4°C. The supernatant was used as the viral stock. Viral B2 gene was amplified using one set of primers (CGCAATGAAGGATGTCTGG and GTGTGGGTGCTCCTAAGA) and confirmed with Sanger sequencing. The amplicon revealed a mutated start codon in the B2 sequence, along with the insertion of a stop codon at the 58th amino acid position of the B2 gene.

### Cell culture

*Drosophila* S2 cells were maintained at 25°C in Schneider’s Insect Medium (Sigma) supplemented with 10% heat-inactivated fetal bovine serum (LONSERA), 5 mM Sodium Bicarbonate (Sigma), 5 mM Calcium Chloride (Sigma), and 100 U/mL penicillin-streptomycin (GBICO).

### Gene knockdown by RNAi

Cell-based RNAi using the bathing method were carried out as described previously. First, primers containing the T7 promoter at their 5' end for the indicated genes were designed and synthesized, and their sequences are presented in Table S2. Then, dsRNAs of each gene were synthesized from DNA templates obtained by PCR with sets of primers using T7 transcription kit (Vazyme). In 12-well plate, 400 µL of serum-free medium containing 4 × 10^6^ cells/mL were bathed with 7 µg of dsRNA at 25°C for 30 min. The soaked cells were then supplemented with a complete medium to make the final concentration of FBS to 10%. Cells were incubated for 2–3 days at 25°C to obtain the indicated genes knockdown cells for further experiments and analyses.

### Transfection of plasmids

*Drosophila* S2 cells were seeded in plates or flasks and grown for 4 h to be 70%–80% confluent. Then, 1 µg or 6 µg plasmids were transfected into cells in 12-well plates or T25 flasks, respectively, using Lipofectamine 3000 Transfection Reagent (Invitrogen) according to the manufacturer’s instructions. After incubation at 25°C for 24 h, cells were stimulated with 25 mM CuSO_4_ for an additional 24 h. Transfected cells were directly harvested for RNA and protein analysis or used for further experiments.

### Reverse transcription-quantitative PCR (RT-qPCR)

Total RNA was extracted from S2 cells harvested in each assay using TRIzol (TIANGEN). The RNA was then treated with DNase I (Thermo Fisher Scientific) to remove genomic DNA contamination. Next, reverse transcription was performed with the primer mix containing random primer and oligo dT primer using the RevertAid First Strand cDNA Synthesis Kit (Fermentas, USA) according to the manufacturer’s instructions. Reverse transcription-quantitative PCR (RT-qPCR) was performed using ChamQ Universal SYBR qPCR Master Mix (Vazyme) and QuantStudio 7 Flex system (Life Technologies). The RT-qPCR program was as follows: 95°C for 3 min; 40 cycles of 95°C for 30 s, and 60°C for 30 s. The expression level for target genes versus the control *rp49* was calculated using the 2^-ΔΔCt^ method (50). The primer sequences used for RT-qPCR are listed in Table S2.

### Co-immunoprecipitation (Co-IP) and LC-MS/MS

S2 cells were co-transfected with pMT-GFP-B2 and pMT-V5-CDK12 plasmids and collected 48 h late. Co-IP assays were carried out as described in our previous report (21). Briefly, 5 × 10^7^ cells were suspended in RIPA buffer (50 mM Tris-HCl [pH 7.4], 150 mM NaCl, 5 mM MgCl_2_, 1 mM EDTA, 10% glycerol, 1% Nonidet P-40, and 1 mM PMSF) on ice for 30 min and mixed every 10 min. After centrifugation at 13,000 rpm for 10 min at 4°C, the lysates were pre-clear by incubating with 50 µL protein A agarose for 1 h. Protein A agarose was washed three times with RIPA buffer and then coupled with anti-V5 tag antibody (Invitrogen) or anti-GFP antibody (Santa Cruz) for 1 h at 4°C. The pre-clear lysates were incubated with antibody-beads complex at 4°C overnight. The beads were washed five times with RIPA buffer. The proteins were eluted from the beads by boiling in 1× SDS loading buffer for 5 min and further analyzed by western blot.

S2 cells were transfected with pMT-V5-B2 plasmids and collected 48 h late. The cell lysates were followed by Co-IP assay with anti-V5 tag antibody as described above. The proteins were digested with trypsin on the beads and then sent to Beijing BiotechPack Scientific (China) for LC-MS/MS.

### Flow cytometry and cell sorting

S2 cells were pretreated with double-stranded RNAs (dsRNAs) targeting CDK12 or control dsRNA (β*-galactosidase*, β*-gal*) for 48 h. Following this, the cells were transfected with either pMT-B2-GFP or pMT-GFP plasmids for an additional 48 h. After the transfection period, GFP-positive cells were isolated using a FACS Aria III cell sorter (BD Biosciences) set at 85 psi (pounds per square inch). Cell sorting was performed by trained students at the Flow Core Facility of Hangzhou Normal University. The GFP+ cells were sorted based on defined phenotypic characteristics, achieving a purity of greater than 95%.

### Western blot analysis

For western blot, the total protein extracts and the protein from Co-IP assays were separated by 10% (wt/vol) sodium dodecyl sulfate-polyacrylamide gel electrophoresis (SDS-PAGE) and transferred to PVDF membrane (Millipore) at 200 mA for 90 to 120 min. Membranes were blocked with 5% non-fat dry milk in Tris-buffered saline with 0.05% Tween 20 Detergent (TBST) at room temperature for 1 h and then incubated with anti-V5 tag antibody (1:10000, Invitrogen, R960-25), anti-GFP (1:4000, Proteintech, 50430–2-AP) antibody, anti-CDK12 (generated by HuaAn Biotechnology Company, Hangzhou, China), or anti-GAPDH (1:20000, Proteintech, 60004) antibody, respectively, at 4°C overnight. After washing five times, the membranes were incubated with HRP-labeled goat anti-mouse IgG secondary antibody (Beyotime, 1:5000) at room temperature for 1 h. The final detection was performed using the BeoECL plus chemiluminescence substrate (Beyotime) and analyzed with Fusion FX SPECTRA (VILBER).

### Dual luciferase report assay

CDK12 knockdown cells and wild-type cells were co-transfected with psiCHECK-2 vector, which encodes *firefly* luciferase and *Renilla* luciferase, and pMT-B2-V5 vector or pMT vector. At 24 h post-transfection, the cells were treated with dsRNA targeting the *firefly* luciferase. The *firefly* luciferase and *Renilla* luciferase activities were measured using the dual-luciferase reporter assay system (Promega) according to the manufacturer’s instructions. The relative *firefly* luciferase activities were normalized to *Renilla* luciferase activity and wild-type cells group expressing B2 protein, in which the *Renilla* luciferase activity served as a transfection control.

### Small RNA library construction

Total RNAs were isolated from CDK12 knockdown, and wild-type cells were infected with FHV for 24 h using TRIzol (TIANGEN). Small RNA libraries were prepared using NEBNext Multiplex Small RNA Library Prep Set for Illumina (NEB) according to the manufacturer’s guide. Briefly, 2 µg total RNA was ligated to 3' and 5' adapters, followed by reverse transcription into cDNA. The cDNAs were amplified by PCR and subjected to 8% polyacrylamide gel to recover the products ranging from 140 to 160 bp. The library quality was assessed on the Agilent Bioanalyzer 2100 system. Qualified small RNA libraries were sequenced on an Illumina Hiseq 2500 platform.

### Small RNA-seq analysis

Raw small RNA reads were first processed to remove the 3' adapter and contaminants formed through adapter-adapter ligation using the cutadapt program. Low-quality bases were cut using Trimmomatic software, and read lengths between 17 and 28 nt were used for subsequent analyses. The clean reads were mapped to either FHV RNA1 (GenBank accession no. NC_004146) and FHV RNA2 (GenBank accession no. NC_004144) with BLASTN. Only reads that are 100% identical or complementary to FHV RNA1 or RNA2 were kept for further analyses.

## SUPPLEMENTAL MATERIAL

Figure S1 mbio.02868-24-s0001.tif DCV-1A protein has function similar to B2 but does not interact with host CDK12.

Legend mbio.02868-24-s0002.docx Legend for Fig. S1.

Captions mbio.02868-24-s0003.docx Captions for Tables S1 and S2.

Table S1 mbio.02868-24-s0004.xlsx MS raw data.

Table S2 mbio.02868-24-s0005.xlsx Primer list.

ASM does not own the copyrights to Supplemental Material that may be linked to, or accessed through, an article. The authors have granted ASM a non-exclusive, world-wide license to publish the Supplemental Material files. Please contact the corresponding author directly for reuse.

## References

[1] Guo Z, Li Y, Ding SW. 2019. Small RNA-based antimicrobial immunity. Nat Rev Immunol 19:31–44. doi:10.1038/s41577-018-0071-x 30301972

[2] Ding SW. 2010. RNA-based antiviral immunity. Nat Rev Immunol 10:632–644. doi:10.1038/nri2824 20706278

[3] Bronkhorst AW, van Rij RP. 2014. The long and short of antiviral defense: small RNA-based immunity in insects. Curr Opin Virol 7:19–28. doi:10.1016/j.coviro.2014.03.010 24732439

[4] Marques JT, Wang J-P, Wang X, de Oliveira KPV, Gao C, Aguiar E, Jafari N, Carthew RW. 2013. Functional specialization of the small interfering RNA pathway in response to virus infection. PLoS Pathog 9:e1003579. doi:10.1371/journal.ppat.1003579 24009507 PMC3757037

[5] Csorba T, Kontra L, Burgyán J. 2015. Viral silencing suppressors: tools forged to fine-tune host-pathogen coexistence. Virology (Auckl) 479–480:85–103. doi:10.1016/j.virol.2015.02.028 25766638

[6] Wu Q, Wang X, Ding SW. 2010. Viral suppressors of RNA-based viral immunity: host targets. Cell Host Microbe 8:12–15. doi:10.1016/j.chom.2010.06.009 20638637 PMC2929401

[7] Bonning BC, Saleh MC. 2021. The interplay between viruses and RNAi pathways in insects. Annu Rev Entomol 66:61–79. doi:10.1146/annurev-ento-033020-090410 33417818

[8] Ferrero DS, Busnadiego I, Garriga D, Guerra P, Martín MT, Kremer L, Usón I, Rodriguez JF, Verdaguer N. 2021. Structure and dsRNA-binding activity of the Birnavirus Drosophila X Virus VP3 protein. J Virol 95:e02166-20. doi:10.1128/JVI.02166-20 33239452 PMC7851550

[9] van Rij RP, Saleh M-C, Berry B, Foo C, Houk A, Antoniewski C, Andino R. 2006. The RNA silencing endonuclease Argonaute 2 mediates specific antiviral immunity in Drosophila melanogaster. Genes Dev 20:2985–2995. doi:10.1101/gad.1482006 17079687 PMC1620017

[10] Fareh M, van Lopik J, Katechis I, Bronkhorst AW, Haagsma AC, van Rij RP, Joo C. 2018. Viral suppressors of RNAi employ a rapid screening mode to discriminate viral RNA from cellular small RNA. Nucleic Acids Res 46:3187–3197. doi:10.1093/nar/gkx1316 29325071 PMC5888754

[11] Nayak A, Kim DY, Trnka MJ, Kerr CH, Lidsky PV, Stanley DJ, Rivera BM, Li KH, Burlingame AL, Jan E, Frydman J, Gross JD, Andino R. 2018. A viral protein restricts Drosophila RNAi immunity by regulating argonaute activity and stability. Cell Host Microbe 24:542–557. doi:10.1016/j.chom.2018.09.006 30308158 PMC6450077

[12] Nayak A, Berry B, Tassetto M, Kunitomi M, Acevedo A, Deng C, Krutchinsky A, Gross J, Antoniewski C, Andino R. 2010. Cricket paralysis virus antagonizes Argonaute 2 to modulate antiviral defense in Drosophila. Nat Struct Mol Biol 17:547–554. doi:10.1038/nsmb.1810 20400949 PMC3815677

[13] Watanabe M, Iwakawa HO, Tadakuma H, Tomari Y. 2017. Biochemical and single-molecule analyses of the RNA silencing suppressing activity of CrPV-1A. Nucleic Acids Res 45:10837–10844. doi:10.1093/nar/gkx748 28977639 PMC5737572

[14] Aliyari R, Wu Q, Li H-W, Wang X-H, Li F, Green LD, Han CS, Li W-X, Ding S-W. 2008. Mechanism of induction and suppression of antiviral immunity directed by virus-derived small RNAs in Drosophila. Cell Host Microbe 4:387–397. doi:10.1016/j.chom.2008.09.001 18854242 PMC2584229

[15] Lingel A, Simon B, Izaurralde E, Sattler M. 2005. The structure of the flock house virus B2 protein, a viral suppressor of RNA interference, shows a novel mode of double-stranded RNA recognition. EMBO Rep 6:1149–1155. doi:10.1038/sj.embor.7400583 16270100 PMC1369214

[16] Seo JK, Kwon SJ, Rao ALN. 2012. Molecular dissection of Flock house virus protein B2 reveals that electrostatic interactions between N-terminal domains of B2 monomers are critical for dimerization. Virology (Auckl) 432:296–305. doi:10.1016/j.virol.2012.05.023 22721960

[17] Lu R, Maduro M, Li F, Li HW, Broitman-Maduro G, Li WX, Ding SW. 2005. Animal virus replication and RNAi-mediated antiviral silencing in Caenorhabditis elegans. Nature New Biol 436:1040–1043. doi:10.1038/nature03870 PMC1388260 16107851

[18] Chao JA, Lee JH, Chapados BR, Debler EW, Schneemann A, Williamson JR. 2005. Dual modes of RNA-silencing suppression by Flock House virus protein B2. Nat Struct Mol Biol 12:952–957. doi:10.1038/nsmb1005 16228003

[19] Singh G, Popli S, Hari Y, Malhotra P, Mukherjee S, Bhatnagar RK. 2009. Suppression of RNA silencing by Flock house virus B2 protein is mediated through its interaction with the PAZ domain of Dicer. FASEB J 23:1845–1857. doi:10.1096/fj.08-125120 19193719

[20] Betting V, Van Rij RP. 2020. Countering counter-defense to antiviral RNAi. Trends Microbiol 28:600–602. doi:10.1016/j.tim.2020.05.018 32534913

[21] Zhang L, Xu W, Gao X, Li W, Qi S, Guo D, Ajayi OE, Ding S-W, Wu Q. 2020. lncRNA sensing of a viral suppressor of RNAi activates non-canonical innate immune signaling in Drosophila. Cell Host Microbe 27:115–128. doi:10.1016/j.chom.2019.12.006 31917956

[22] Bartkowiak B, Liu P, Phatnani HP, Fuda NJ, Cooper JJ, Price DH, Adelman K, Lis JT, Greenleaf AL. 2010. CDK12 is a transcription elongation-associated CTD kinase, the metazoan ortholog of yeast Ctk1. Genes Dev 24:2303–2316. doi:10.1101/gad.1968210 20952539 PMC2956209

[23] Hsin JP, Manley JL. 2012. The RNA polymerase II CTD coordinates transcription and RNA processing. Genes Dev 26:2119–2137. doi:10.1101/gad.200303.112 23028141 PMC3465734

[24] Davidson L, Muniz L, West S. 2014. 3’ end formation of pre-mRNA and phosphorylation of Ser2 on the RNA polymerase II CTD are reciprocally coupled in human cells. Genes Dev 28:342–356. doi:10.1101/gad.231274.113 24478330 PMC3937513

[25] Li X, Chatterjee N, Spirohn K, Boutros M, Bohmann D. 2016. Cdk12 is a gene-selective RNA polymerase II kinase that regulates a subset of the transcriptome, including Nrf2 target genes. Sci Rep 6:21455. doi:10.1038/srep21455 26911346 PMC4766476

[26] Blazek D, Kohoutek J, Bartholomeeusen K, Johansen E, Hulinkova P, Luo Z, Cimermancic P, Ule J, Peterlin BM. 2011. The Cyclin K/Cdk12 complex maintains genomic stability via regulation of expression of DNA damage response genes. Genes Dev 25:2158–2172. doi:10.1101/gad.16962311 22012619 PMC3205586

[27] Pan L, Xie W, Li K-L, Yang Z, Xu J, Zhang W, Liu L-P, Ren X, He Z, Wu J, Sun J, Wei H-M, Wang D, Xie W, Li W, Ni J-Q, Sun F-L. 2015. Heterochromatin remodeling by CDK12 contributes to learning in Drosophila. Proc Natl Acad Sci U S A 112:13988–13993. doi:10.1073/pnas.1502943112 26508632 PMC4653196

[28] Zhang X, Nguyen KD, Rudnick PA, Roper N, Kawaler E, Maity TK, Awasthi S, Gao S, Biswas R, Venugopalan A, Cultraro CM, Fenyö D, Guha U. 2019. Quantitative mass spectrometry to interrogate proteomic heterogeneity in metastatic lung adenocarcinoma and validate a novel somatic mutation CDK12-G879V. Mol Cell Proteomics 18:622–641. doi:10.1074/mcp.RA118.001266 30617155 PMC6442362

[29] Nguyen B, Mota JM, Nandakumar S, Stopsack KH, Weg E, Rathkopf D, Morris MJ, Scher HI, Kantoff PW, Gopalan A, Zamarin D, Solit DB, Schultz N, Abida W. 2020. Pan-cancer analysis of CDK12 alterations identifies a subset of prostate cancers with distinct genomic and clinical characteristics. Eur Urol 78:671–679. doi:10.1016/j.eururo.2020.03.024 32317181 PMC7572747

[30] Sokol ES, Pavlick D, Frampton GM, Ross JS, Miller VA, Ali SM, Lotan TL, Pardoll DM, Chung JH, Antonarakis ES. 2019. Pan-cancer analysis of CDK12 loss-of-function alterations and their association with the focal tandem-duplicator phenotype. Oncol 24:1526–1533. doi:10.1634/theoncologist.2019-0214 PMC6975947 31292271

[31] Liu H, Liu K, Dong Z. 2021. Targeting CDK12 for cancer therapy: function, mechanism, and drug discovery. Cancer Res 81:18–26. doi:10.1158/0008-5472.CAN-20-2245 32958547

[32] Dubbury SJ, Boutz PL, Sharp PA. 2018. CDK12 regulates DNA repair genes by suppressing intronic polyadenylation. Nat New Biol 564:141–145. doi:10.1038/s41586-018-0758-y PMC6328294 30487607

[33] Qi N, Zhang L, Qiu Y, Wang Z, Si J, Liu Y, Xiang X, Xie J, Qin C-F, Zhou X, Hu Y. 2012. Targeting of dicer-2 and RNA by a viral RNA silencing suppressor in Drosophila cells. J Virol 86:5763–5773. doi:10.1128/JVI.07229-11 22438534 PMC3347268

[34] Zhang Q, Zhang L, Gao X, Qi S, Chang Z, Wu Q. 2015. DIP1 plays an antiviral role against DCV infection in Drosophila melanogaster. Biochem Biophys Res Commun 460:222–226. doi:10.1016/j.bbrc.2015.03.013 25770426

[35] Nakahara KS, Masuta C, Yamada S, Shimura H, Kashihara Y, Wada TS, Meguro A, Goto K, Tadamura K, Sueda K, Sekiguchi T, Shao J, Itchoda N, Matsumura T, Igarashi M, Ito K, Carthew RW, Uyeda I. 2012. Tobacco calmodulin-like protein provides secondary defense by binding to and directing degradation of virus RNA silencing suppressors. Proc Natl Acad Sci U S A 109:10113–10118. doi:10.1073/pnas.1201628109 22665793 PMC3382489

[36] Chen L, Yan Z, Xia Z, Cheng Y, Jiao Z, Sun B, Zhou T, Fan Z. 2017. A violaxanthin deepoxidase interacts with a viral suppressor of RNA silencing to inhibit virus amplification. Plant Physiol 175:1774–1794. doi:10.1104/pp.17.00638 29021224 PMC5717725

[37] Pumplin N, Voinnet O. 2013. RNA silencing suppression by plant pathogens: defence, counter-defence and counter-counter-defence. Nat Rev Microbiol 11:745–760. doi:10.1038/nrmicro3120 24129510

[38] Yan Y, Tang YD, Zheng C. 2022. When cyclin-dependent kinases meet viral infections, including SARS-CoV-2. J Med Virol 94:2962–2968. doi:10.1002/jmv.27719 35288942 PMC9088476

[39] Zheng C, Tang YD. 2022. The emerging roles of the CDK/cyclin complexes in antiviral innate immunity. J Med Virol 94:2384–2387. doi:10.1002/jmv.27554 34964486

[40] Berro R, Pedati C, Kehn-Hall K, Wu W, Klase Z, Even Y, Genevière A-M, Ammosova T, Nekhai S, Kashanchi F. 2008. CDK13, a new potential human immunodeficiency virus type 1 inhibitory factor regulating viral mRNA splicing. J Virol 82:7155–7166. doi:10.1128/JVI.02543-07 18480452 PMC2446983

[41] Yang BK, Chen J, Teng YC. 2021. CDK12 promotes cervical cancer progression through enhancing macrophage infiltration. J Immunol Res 2021:1–14. doi:10.1155/2021/6645885 PMC7892235 33628849

[42] Juan HC, Lin Y, Chen HR, Fann MJ. 2016. Cdk12 is essential for embryonic development and the maintenance of genomic stability. Cell Death Differ 23:1038–1048. doi:10.1038/cdd.2015.157 26658019 PMC4987723

[43] Weeks SA, Shield WP, Sahi C, Craig EA, Rospert S, Miller DJ. 2010. A targeted analysis of cellular chaperones reveals contrasting roles for heat shock protein 70 in flock house virus RNA replication. J Virol 84:330–339. doi:10.1128/JVI.01808-09 19828623 PMC2798444

[44] Weeks SA, Miller DJ. 2008. The heat shock protein 70 cochaperone YDJ1 is required for efficient membrane-specific flock house virus RNA replication complex assembly and function in Saccharomyces cerevisiae. J Virol 82:2004–2012. doi:10.1128/JVI.02017-07 18057252 PMC2258711

[45] Castorena KM, Weeks SA, Stapleford KA, Cadwallader AM, Miller DJ. 2007. A functional heat shock protein 90 chaperone is essential for efficient flock house virus RNA polymerase synthesis in Drosophila cells. J Virol 81:8412–8420. doi:10.1128/JVI.00189-07 17522196 PMC1951356

[46] Kopek BG, Perkins G, Miller DJ, Ellisman MH, Ahlquist P. 2007. Three-dimensional analysis of a viral RNA replication complex reveals a virus-induced mini-organelle. PLoS Biol 5:e220. doi:10.1371/journal.pbio.0050220 17696647 PMC1945040

[47] Vodovar N, Goic B, Blanc H, Saleh MC. 2011. In silico reconstruction of viral genomes from small RNAs improves virus-derived small interfering RNA profiling. J Virol 85:11016–11021. doi:10.1128/JVI.05647-11 21880776 PMC3194930

[48] Han Y-H, Luo Y-J, Wu Q, Jovel J, Wang X-H, Aliyari R, Han C, Li W-X, Ding S-W. 2011. RNA-based immunity terminates viral infection in adult Drosophila in the absence of viral suppression of RNA interference: characterization of viral small interfering RNA populations in wild-type and mutant flies. J Virol 85:13153–13163. doi:10.1128/JVI.05518-11 21957285 PMC3233157

[49] Seo JK, Kwon SJ, Rao ALN. 2012. A physical interaction between viral replicase and capsid protein is required for genome-packaging specificity in an RNA virus. J Virol 86:6210–6221. doi:10.1128/JVI.07184-11 22438552 PMC3372179

[50] Livak KJ, Schmittgen TD. 2001. Analysis of relative gene expression data using real-time quantitative PCR and the 2^−ΔΔCT^ method. Methods 25:402–408. doi:10.1006/meth.2001.1262 11846609

