# Supplementary material for: CDK12 antagonizes a viral suppressor of RNAi to modulate antiviral RNAi in Drosophila
Source: mBio. 2024 Nov 27;16(1):e02868-24. doi: 10.1128/mbio.02868-24 (PMC11708023; doi:10.1128/mbio.02868-24)
Supplement: Legend — for Fig. S1. [file mbio.02868-24-s0002.docx]

**Figure S1.** **DCV-1A protein function is similar to B2, but not interact with host CDK12. (A)** S2 cells were pretreated with three different dsRNAs targeting CDK12 or control dsRNA (β-gal) for 48 h and transfected with pMT-B2-GFP or pMT-DCV-1A-GFP plasmids for another 48 h. The pretreated cells were infected with FHV△B2, and total RNA was isolated at 24 hpi. The FHV RNA1 levels, relative to the control in B2-V5 group, were determined by RT‒qPCR (top) and protein detection by western blot (bottom). **(B)** S2 cells co-transfected with pMT-V5-CDK12 plasmid and pMT-DCV-1A-GFP plasmids were subjected to Co-IP using anti-V5 and western blot for the immunoprecipitants using the indicated antibodies. Error bars represent SD in three independent experiments performed in triplicate. Statistical analysis was performed using ANOVA; ∗∗∗p < 0.001.
