# Supplementary material for: CDK12 antagonizes a viral suppressor of RNAi to modulate antiviral RNAi in Drosophila
Source: mBio. 2024 Nov 27;16(1):e02868-24. doi: 10.1128/mbio.02868-24 (PMC11708023; doi:10.1128/mbio.02868-24)
Supplement: Captions — for Tables S1 and S2. [file mbio.02868-24-s0003.docx]

**Supplemental Table Legends**

**Table S1.** List of B2-interacting proteins identified by LC-MS/MS analysis in S2 cells.

**Table S2.** Sequences of primers used in this study.
